# Preoperative evaluation of residual tumor in patients with endometrial carcinoma by using ^18^F-FDG PET/CT

**DOI:** 10.7150/jca.39423

**Published:** 2020-02-10

**Authors:** Chunhua Wu, Ruohua Chen, Xiang Zhou, Qian Xia, Jianjun Liu

**Affiliations:** 1 Department of Nuclear Medicine, Ren Ji Hospital, School of Medicine, Shanghai Jiao Tong University, Shanghai, China; 2 Department of Ultrasound, Ren Ji Hospital, School of Medicine, Shanghai Jiao Tong University, Shanghai, China

**Keywords:** PET/CT, endometrial cancer, SUVmax, residual tumor

## Abstract

**Purpose**: To evaluate the value of ^18^F-FDG positron emission tomography (PET)/computed tomography (CT) for determining the presence of residual tumors after curettage in patients with endometrial cancer.

**Methods**: Preoperative ^18^F-FDG PET/CT was performed in 90 women with endometrial cancer. PET/CT parameters and clinical characteristics were compared between patients with and without residual tumors. The clinical characteristics of patients with residual tumors that showed low ^18^F-FDG uptake were also analyzed.

**Results**: Among the 90 patients, 86 had residual tumors. ROC analysis identified a lesion SUVmax value of 5.0 as the optimal cut-off value for predicting whether or not patients had a residual tumor. With the SUVmax cut-off of 5, the sensitivity, specificity, positive predictive value, and negative predictive values for residual tumor prediction were 87.2%, 100%, 100%, and 26.7%, respectively. Univariate analysis showed significant associations between the high PET group (SUVmax > 5) and low PET group (SUVmax ≤ 5), and histologic type (P = 0.043) and tumor size (P < 0.001) in patients with residual tumors. In patients with low-grade and clear cell carcinomas and a tumor size < 1.35 cm, the probability of being in the low-PET group was 47.6%. In such patients, major parts of the residual tumors showed low ^18^F-FDG uptake, similar to that in patients with no residual tumors.

**Conclusion**: SUVmax was the lone predictive value for the presence of residual tumors after curettage in patients with endometrial cancer. Lesion SUVmax greater than 5 suggested a high possibility of residual tumors. In patients with low-grade and clear cell carcinomas with tumor size < 1.35 cm, residual tumors may present low ^18^F-FDG uptake, mimicking the metabolic phenotypes of patients without residual tumors.

## Introduction

Endometrial cancer is one of the most common gynecological malignant tumors worldwide 1,2. Radical treatment remains the main therapeutic approach for endometrial cancer 3,4. Although most patients with endometrial cancer are elderly, some are younger. Younger patients may be treated with fertility-preserving therapies instead of surgery 5,6. Therefore, an accurate assessment of whether or not these patients have residual tumors after curettage or fertility-preserving therapies is of great importance. Residual tumors can be confirmed by hysteroscopy and curettage 7; however, these methods are invasive. Thus, effective, alternative noninvasive strategies that can determine the presence of residual tumors are needed.

^18^F-18 fluorodeoxyglucose (FDG) positron emission tomography (PET) has been widely used to differentiate between malignant tumors and benign lesions 8,9. The maximum standardized uptake value (SUVmax) is a common parameter for predicting malignant tumors 10. Several studies have addressed the correlation between histological factors and SUVmax in endometrial cancer 11-14. However, so far, few studies have examined the role of ^18^F-FDG PET/CT in determining the presence of residual tumors after curettage in patients with endometrial carcinoma. In the current study, we evaluated the relationship between SUVmax of the lesion and the presence of residual tumors. We also analyzed the clinicopathologic features of patients with residual tumors that showed low 18F-FDG uptake, similar to the metabolic phenotypes of patients without residual tumors.

## Materials and Methods

### Study population

Ninety women with endometrial cancer were included in our study. Before ^18^F-FDG PET-CT scans were obtained, endometrial cancer was confirmed in 88 patients by using curettage, hysteroscopy, or colposcopy. Endometrial cancer was confirmed in the remaining two patients by postoperative pathology. All patients undergone ^18^F-FDG PET-CT scans before radical treatment at RenJi Hospital affiliated to the Shanghai Jiaotong University from January 2016 to April 2019. Patients were eligible for inclusion if 1 they had been treated by hysterectomy with lymphadenectomy; 2 they had been confirmed by a histopathologic examination of surgical specimens; 3 adjuvant therapy were not administered before PET-CT scans; and 4 complete case records, including age, menopause, biopsy method, FIGO stage, and the time from biopsy to scan, tumor size, and histologic type, were available. Ninety patients fulfilled the eligibility criteria. The Institutional Review Board of Ren Ji Hospital approved this study, and the informed consent was waived.

### PET-CT

^18^F-FDG PET-CT was conducted using a PET-CT scanner (Biograph mCT, Siemens Systems). All patients received an intravenous injection of 3.7 MBq/kg of ^18^F-FDG after fasting for 6 hours and resting for 1 hour. The mean uptake time was 50 minutes. CT was performed on the same scanner. The CT scan data was obtained using a standardized protocol involving 140 mA, 120 kV, and section thickness of 5.0 mm.

Irregular regions of interest were placed over the area with the most intense ^18^F-FDG uptake. The SUV_max_ was calculated using the formula as follows: maximum pixel value with the region of interest activity (MBq/mL)/(injected dose [MBq]/body weight [kg]). Two experienced nuclear medicine physicians evaluated the PET-CT image.

### Pathological evaluation

When malignant tumor was found by postoperative pathology, the patient was considered to have residual tumor. When malignant tumor was not found by postoperative pathology, the patient was considered to have no residual tumor. Among the 88 patients in whom endometrial cancer was confirmed using curettage, hysteroscopy, or colposcopy before ^18^F-FDG PET-CT, 84 had residual tumors and 4 did not after hysterectomy. The primary and residual tumors were identical in histologic type and differentiation status. With regard to the two patients in whom endometrial cancer was suspected before ^18^F-FDG PET-CT, the cancer was confirmed using surgical specimens. Tumor-related parameters were recorded, including FIGO stage, tumor histological type, maximum tumor size, cervical stromal invasion, depth of myometrial invasion, and lymph node metastasis according to the WHO classification 15.

### Statistical analysis

The data are presented as mean±SD values. Statistically significant differences were compared using the Mann-Whitney *U* test, chi-square test, or Fisher's exact test, where applicable. Statistical analyses were conducted using SPSS, version 13.0 (SPSS Inc.).

## Results

### Patient characteristics

The patient characteristics are shown in Table 1. 90 women (mean age, 55.5 years; range, 28-83 years) were consisted in this study. Fifty-six patients are already menopausal. Before ^18^F-FDG PET/CT scans, endometrial cancer was confirmed in 16 patients were confirmed by hysteroscopy or colposcopy and in 72 patients by curettage; the remaining two were suspected to have endometrial cancer, but the disease could not be confirmed by pathological assessments. The mean time from biopsy to the ^18^F-FDG PET/CT scan was 17.5 days. SUVmax values of the lesions ranged between 2 and 33.2; the average value was 6.34. Among the 90 patients, 86 had residual tumors whereas four showed no residual tumors. Among the 86 cases with residual tumors, 27 showed grade 1 carcinoma, 42 showed grade 2 carcinoma, eight showed grade 3 carcinoma, and three cases each showed serous carcinoma, clear cell carcinoma, and endometrioid carcinoma with phosphatization. Among the four patients with no residual tumors, curettage confirmed the presence of endometrioid tumors but no residual tumors were observed after radical treatment of endometrial cancer.

### Differences between patients with and without residual tumors

**Table 2** depicts F-18 FDG PET/CT imaging data and patient characteristics grouped on the basis of the presence of residual tumors (n = 90). No significant intergroup differences were found in terms of age, menopause, biopsy method, FIGO stage, and the time from biopsy to scan. However, significant intergroup differences were found with respect to the SUVmax of the lesion. More specifically, patients with residual tumors showed significantly higher SUVmax values than those with no residual tumors (12.2 ± 6.8 vs. 3.6 ± 1.4; P < 0.001). Data for the four patients who had no residual tumors are listed in Table 3.

### Measurement of SUVmax cut-off value

**Figure 1:**
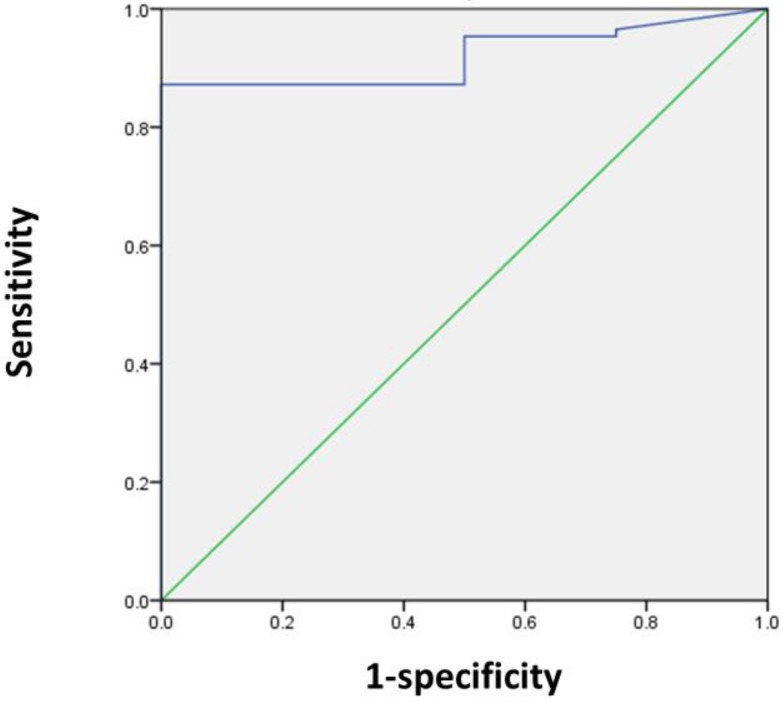
** ROC curve analysis for differentiating patients with and without residual tumors according to the SUVmax in 90 patients.** The area under the curve was 0.92 (95% CI 0.851-0.989, P = 0.005), and 5 was determined as the best SUVmax value for predicting the presence of residual tumors. With an SUVmax of 5 as the threshold, the sensitivity, specificity, positive predictive value, and negative predictive value for the prediction of tumor residues were 87.2%, 100%, 100%, and 26.7%, respectively.

Next, we sought to determine the SUVmax threshold for optimal differentiation of patients with and without residual tumors. ROC curve analysis revealed that the highest accuracy (87.8%) was obtained with an SUVmax cut-off of 5 and the area under the curve was 0.920 ± 0.035 (Figure 1). With an SUVmax cut-off of 5, the sensitivity, specificity, positive predictive value, and negative predictive value for the prediction of residual tumors were 87.2% (75/86), 100% (4/4), 100% (75/75), and 26.7% (4/15), respectively.

### The relationships between clinicopathologic features and PET/CT results in patients with residual tumors

Of 90 patients, 86 patients had residual tumors. We further evaluated the associations between clinicopathologic features and PET/CT results in these 86 patients by univariate analysis (Table 4). The patients were divided into the following groups according to the SUVmax of the tumors: high-PET group (SUVmax > 5) and low-PET group (SUVmax ≤ 5). No significant intergroup differences were found in terms of age, menopause, biopsy method, the time from biopsy to scan, myometrial invasion, lymph node metastasis, and FIGO stage. However, the two groups showed a significant difference in histologic type (P = 0.043) and tumor size (P < 0.001).

**Figure 2:**
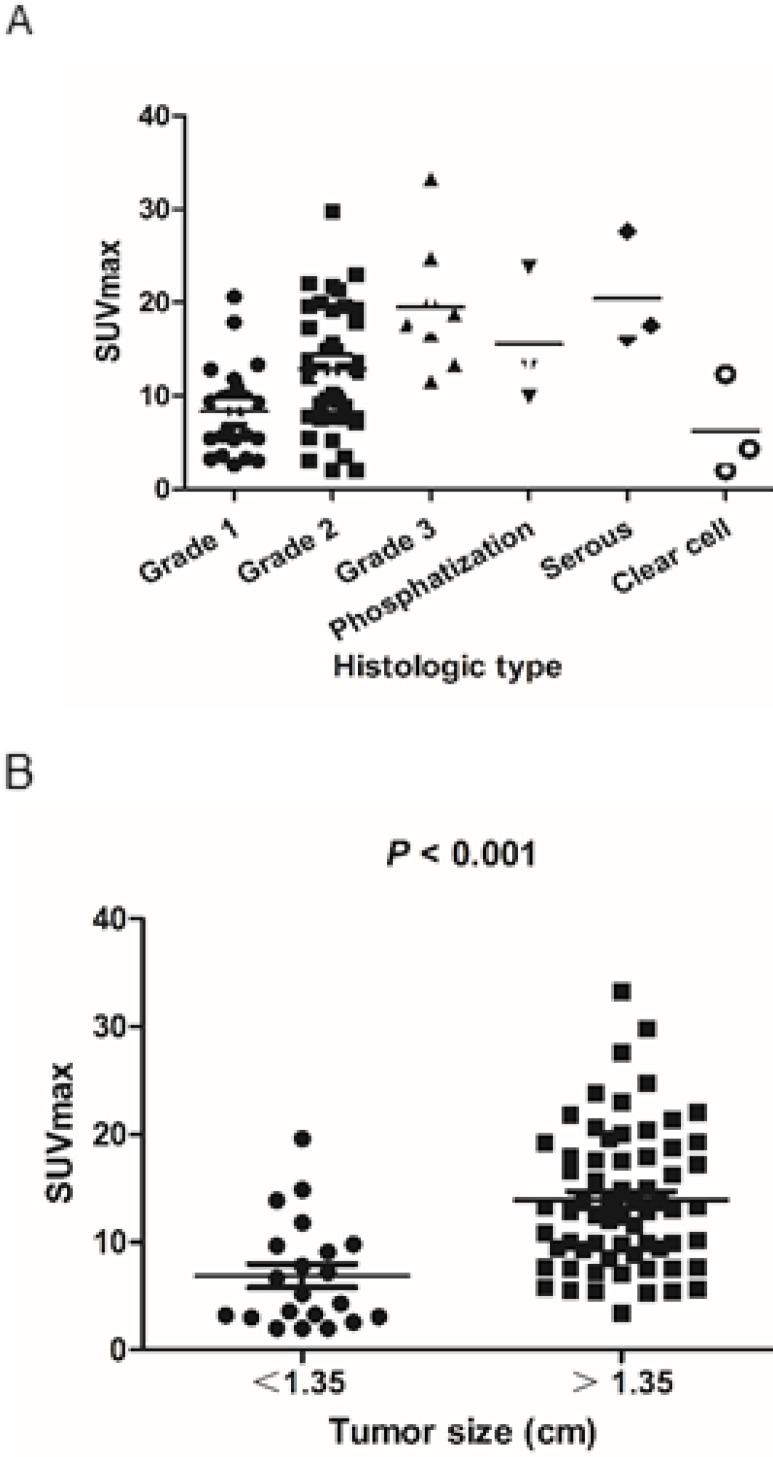
** A)** Scatter plot of the tumor SUVmax according to the pathological subtype and grade. **B)** SUVmax was significantly lower in small tumors (<1.35 cm) than in large tumors (>1.35 cm) (6.9 ± 4.9 and 13.9 ± 6.5, respectively; P < 0.001).

The scatter plot of SUVmax for each endometrioid carcinoma subtype and grade is shown in Figure 2A. SUVmax values of low-grade carcinoma, high-grade carcinoma, adenocarcinoma with phosphatization, serous carcinoma, and clear cell carcinoma were 11.1 ± 6.1, 19.5 ± 6.9, 15.6 ± 7.3, 20.4 ± 6.2, and 6.2 ± 5.4, respectively. The rates of patients in the low-PET group among patients with low-grade cancer (Grade 1 and 2), high-grade cancer (Grade 3), adenocarcinoma with phosphatization, serous carcinoma, and clear cell carcinoma were 13.0% (9/69), 0% (0/8), 0% (0/3), 0% (0/3), and 66.7% (2/3), respectively.

Optimal cut-off values were 1.35 cm for tumor size, as determined by receiver operating characteristic curve analysis for optimal differentiation of patients in the high- and low-PET group. SUVmax was significantly lower in patients with small tumor size (<1.35 cm) than in those with a large tumor size (>1.35 cm) (6.9 ± 4.9 and 13.9 ± 6.5, respectively; P < 0.001) (Figure 2B). Thus, the rate of patients in the low-PET group was significantly higher among those with small tumors (<1.35 cm) than in those with large tumors (>1.35 cm) (47.6% vs. 1.5%, P < 0.001).

### Prediction of patients in the low- or high-PET group among those with residual tumors

Next, using the abovementioned two significant parameters of SUVmax for histologic type and tumor size, we categorized the patients into the following groups based on their potential to be in the low- or high-PET group: high potential in the low-PET group (low-grade and clear cell carcinoma and tumor size < 1.35 cm), moderate-potential group (low grade and clear cell carcinoma and tumor size >1.35cm, or high-grade carcinoma, adenocarcinoma with phosphatization, serous carcinoma and tumor size < 1.35 cm), and low-potential in the low-PET group (high-grade carcinoma, adenocarcinoma with phosphatization, serous carcinoma and tumor size > 1.35 cm). The probability of patients being in the low-PET group was 47.6% (10/21), 2.0% (1/51), and 0% (0/14), respectively (*P* < 0.001; Table 5).

## Discussion

An increasing number of young people have endometrial cancer. They may use hormone therapy and other fertility-preserving treatments rather than radical resection of endometrial cancer. Accurate assessment of residual or recurrent tumors in these patients is of great necessity. In this study, the potential of FDG PET/CT to distinguish patients with and without residual tumors after curettage was analyzed. To our knowledge, this was the first study to assess whether FDG PET/CT could be used for identification of residual tumors in endometrial cancers.

Several studies have evaluated ^18^F-FDG PET in the diagnosis and prognosis of patients with endometrial cancer 16-18. Kazuhiro Kitajima et al. 19 found that the metabolic parameters of ^18^F-FDG PET/CT are useful for differentiating high-risk from low-risk endometrial carcinoma. Takagi et al. 20 found that PET/CT is useful for distinguishing between G1/G2 or higher grade in endometrial cancers. However, few studies have examined whether PET/CT could be used for identification of patients with and without residual tumors. In the current study, we found that the SUVmax was the only factor that could predict the presence of residual tumors in endometrial cancers after curettage. With an SUVmax cut-off of 5, the sensitivity, specificity, positive predictive value, and negative predictive value for the prediction of residual tumors were 87.2%, 100%, 100%, and 26.7%, respectively. Thus, lesions with SUVmax greater than 5 indicate a high possibility of residual tumors.

In our present series, we further analyzed the relationship between clinicopathologic features and SUVmax in patients with residual tumors. We found that histologic type and tumor size were significantly correlated with the SUVmax of residual tumors. We further divided patients with residual tumors into three groups on the basis of their potential for being in the low- or high-PET group. We found that when patients had low-grade and clear cell carcinoma and tumor size < 1.35 cm, their probability of being in the low-PET group was 47.6%. In such patients, a substantial proportion of the residual tumors demonstrated low ^18^F-FDG uptake similar to that in patients with no residual tumors. Thus, for patients with low-grade and clear cell carcinoma and tumor size < 1.35 cm, even if the SUVmax was relatively low (SUVmax < 5) after curettage or hormone therapy, we cannot arbitrarily assume that there is no residual tumor.

Our study was limited by the small sample size and its retrospective design. In addition, it is not possible to establish a cut-off value for SUVmax in the clinical setting. Nonetheless, our study may be useful for the development of noninvasive strategies for predicting residual tumors in patients with endometrial cancer. Future large prospective studies are needed to verify these results.

## Conclusions

Our results were the first to show that F-18 FDG PET/CT could be used for identification of residual tumors in endometrial cancers, and the findings demonstrated that in patients with low-grade and clear cell carcinoma and tumor size < 1.35 cm, the residual tumors may present low ^18^F-FDG uptake, mimicking the metabolic phenotypes of patients with no residual tumor. These results could advance the development of noninvasive methods to predict residual tumors after curettage or hormone therapy in patients with endometrial cancer.

## Figures and Tables

**Table 1:** Patient characteristics (n=90)

Characteristics	No. of Patients
**Age (y)**	
Mean ± SD	55.5±12.0
Range	28-83
**Menopause**	
No	34
Yes	56
**Time from biopsy to scan (Days)**	17.5±18.5
**Biopsy method before scan**	
Hysteroscopy or colposcopy	16
Curettage	72
None	2
**SUVmax**	
Mean ± SD	11.8±6.9
Range	2-33.2
**Tumor Size (mm)**	2.6±1.8
**Residual Tumors**	86
Grade 1	27
Grade 2	42
Grade 3	8
Phosphatization	3
Serous	3
Clear cell	3
**No residual Tumors**	4
Endometrioid	4

**Table 2:** Differences between patients with residual tumors and having no residual tumors (n=90)

Characteristics	Total (n=90)	Residual tumors (n=86)	No residual tumors (n=4)	*χ2*	P value
**Age**		55.3±12.2	59.8±4.4		0.472
**Menopause**					
No	34	34	0	2.54	0.293
Yes	56	52	4		
**Biopsy method**					
Hysteroscopy or colposcopy	16	16	0	1.047	0.593
Curettage	72	68	4		
None	2	2	0		
**FIGO stage**					
1	83	79	4	0.353	0.95
2	1	1	0		
3	5	5	0		
4	1	1	0		
**Time from biopsy to scan**		17.8± 18.8	11.7± 7.5		0.525
**SUVmax**		12.2 ± 6.8	3.6±1.4		0.001

**Table 3:** Details of the four patients with no residual tumors

Case	Age	Menopause	Biopsy method	FIGO stage	Time from biopsy to scan (Days)	Histologic type	SUVmax
1	55	Yes	Curettage	1	7	Endometrioid	2
2	64	Yes	Curettage	1	16	Endometrioid	2.9
3	57	Yes	Curettage	1	4	Endometrioid	4.8
4	63	Yes	Curettage	1	20	Endometrioid	4.7

**Table 4:** Patient Characteristics According to low and high PET group (n=86)

Characteristics	Total (n=86)	Low	High	χ2	P
**Age**		53.8±10.7	55.5±12.4		0.665
**Menopause**					
0	34	4	30	0.053	0.818
1	52	7	45		
**Biopsy method**					
Hysteroscopy or colposcopy	16	1	15	1.131	0.568
Curettage	68	10	58		
None	2	0	2		
**Time from biopsy to scan**		17.6±8.1	17.8±19.0		0.974
**Myometrial invasion**					
<50%	66	10	56	1.418	0.445
≥50%	20	1	19		
**Lymph node metastasis**					
Absent	79	10	69	0.015	0.902
Present	7	1	6		
**Histologic type**					
Grade 1/2	69	9	60	9.864	0.043
Grade 3	8	0	8		
Phosphatization	3	0	3		
Serous	3	0	3		
Clear cell	3	2	1		
**Tumor Size (mm)**		0.6±0.5	2.8±1.8		<0.001
**FIGO stage**					
1	79	10	69	0.015	0.902
2-4	7	1	6		

**Table 5:** Potential to be in the low-PET group for patients with residual tumors, as indicated by histologic type and tumor size (n = 86)

		PET group (%)		
Potential	Total	Low	High	P
**High**	21	47.6%	52.4%	<0.001
**Moderately**	51	2%	98%	
**Low**	14	0%	100%	
